# Ultrasound Detection of Below-the-Knee Medial Arterial Calcifications in Asymptomatic Patients Is an Early Negative Predictor of Major Adverse Cardiovascular Events

**DOI:** 10.3390/diagnostics15172273

**Published:** 2025-09-08

**Authors:** Giulia Baldazzi, Nicola Lamberti, Martina Saladini, Maria Cristina Taddia, Valentina Ficarra, Fabio Manfredini, Aaron Thomas Fargion

**Affiliations:** 1 School of Vascular Surgery, University of Ferrara, 44121 Ferrara, Italy; 2 Department of Neuroscience and Rehabilitation, University of Ferrara, 44121 Ferrara, Italy; nicola.lamberti@unife.it (N.L.); fabio.manfredini@unife.it (F.M.); 3 School of Medicine, Department of Translational Medicine and for Romagna, University of Ferrara, 44121 Ferrara, Italy; martinasaladini9@gmail.com; 4 Unit of Vascular and Endovascular Surgery, Department of Cardio-Thoracic-Vascular Surgery, S. Anna University Hospital of Ferrara, 44124 Ferrara, Italy; cristinataddia@gmail.com (M.C.T.); v.ficarra@ospfe.it (V.F.); aaron.fargion@ospfe.it (A.T.F.); 5 Program of Vascular Rehabilitation and Exercise Medicine, S. Anna University Hospital of Ferrara, 44124 Ferrara, Italy

**Keywords:** tibial artery ultrasound, vascular calcification, medial arterial calcification, major adverse cardiovascular events, asymptomatic peripheral arterial disease

## Abstract

**Background:** Medial arterial calcification (MAC) is a vascular disorder that affects the arterial media layer. It represents a predictor of major adverse limb events in patients affected by diabetes mellitus (DM). This single-center retrospective observational study investigates whether ultrasound (US) detection of MAC in below-the-knee (BTK) vessels represents a negative predictor of major adverse cardiovascular events (MACE) in asymptomatic patients. **Methods:** In 2019, 584 patients, referred to the Vascular Surgery Unit for lower limb US, were examined by the same operator, who assessed the presence of BTK MAC. The primary outcome was the rate of MACE during a 5-year follow-up period. The secondary outcomes included the development of peripheral arterial disease (PAD), the overall survival rates, lower limb revascularizations, and major amputations. **Results:** MAC in BTK vessels was highlighted in 239 patients (MAC+) who exhibited a younger age (*p* < 0.001), DM (*p* < 0.001), and chronic kidney disease (CKD) (*p* = 0.048). The 345 subjects without MAC (MAC−) showed prior myocardial infarction (*p* < 0.001), stroke (*p* = 0.034), and smoking habits (*p* < 0.001). After propensity score matching, the MAC+ group presented a higher risk of MACE (HR: 1.84; CI: 1.01–3.38; *p* = 0.047) during a median follow-up of 57 months. Age (HR: 1.06; CI: 1.01–1.12) and MAC (HR: 1.22; CI: 1.06–1.57) were independently associated with MACE. New diagnoses of PAD mainly occurred in the MAC− group (*p* < 0.001). No differences were observed in major amputations, revascularization procedures, or overall survival rates. **Conclusions:** Ultrasound detection of BTK MAC was associated with the presence of DM and CKD and with a 1.8-fold increased risk of developing a MACE within 5 years in asymptomatic patients.

## 1. Introduction

Medial arterial calcification (MAC) of the lower extremities (LE), also referred to as Mönckeberg Sclerosis, is a chronic vascular disorder that was first characterized by Johann Georg Mönckeberg in 1903, when he described a trace that depicted the vessel all along its route in the first lower limb radiographies [1].

For several years, MAC has been considered an uneventful morphological finding; however, in recent decades, its histological basis has been better defined, leading to considering MAC and atherosclerosis as two separate entities caused by different pathogenic pathways [2]. In particular, the atherosclerotic process primarily affects the intimal layer of the arterial wall, where necrotic foci develop within the plaque, leading to an increase in intima–media thickness. This parameter is typically evaluated during ultrasound (US) scan of the carotid or femoral arteries and its role as an early marker of increased cardiovascular risk has already been established [3]. Calcifications in the medial layer, instead, develop from deposits of calcium phosphate (CaP) in the extracellular matrix (ECM) of the arterial smooth muscle layer [4]. Based on these findings, multiple studies have associated MAC presence with chronic kidney disease (CKD), diabetes mellitus (DM), and aging and established its role as an independent predictor of coronary events [5] and peripheral arterial disease (PAD) development, which are historically related to the atherosclerotic process only [6]. Moreover, MAC detection in below-the-knee (BTK) [7] and/or pedal arteries [8] represent an independent predictor of major adverse limb events, for example major amputations [9], in patients affected by chronic limb-threatening ischemia (CLTI).

In multiple studies, several imaging modalities, such as X-rays (XR) and computed tomography (CT), have been used to detect MAC in the arterial axis of the lower extremities [10]; recently, several authors have adduced Duplex Ultrasound (DUS) scan as a non-invasive and reliable alternative [11].

Nevertheless, the real prevalence of MAC is still not established; both PAD and coronary artery disease (CAD) are characterized by slow progression, with patients remaining asymptomatic for decades [12,13]. Consequently, MAC evaluation takes place only when patients are referred to health care providers in the symptomatic stage.

The aim of the present study is to investigate whether MAC detection could represent a negative predictor of major adverse cardiovascular events (MACE) and clinically established PAD development in a cohort of asymptomatic elderly patients who underwent lower limb DUS scans at a vascular surgery facility.

## 2. Materials and Methods

### 2.1. Study Design and Cohort

This single-center retrospective observational study is reported according to the STROBE guidelines for cohort studies [14] (STROBE flowchart shown in Supplementary Figure S1). The CE-AVEC Ethics Committee approved the study (approval number Oss/277/2019). Owing to the nature of the study, written informed consent was waived by the Local Ethics Committee. We analyzed all medical records of males and females aged >18 years, referred by the general practitioner or other specialists to the Unit of Vascular and Endovascular Surgery at the University Hospital of Ferrara for an evaluation of lower limb arteries through ultrasound scans for heterogenous diagnostic indications (e.g., nonspecific lower limbs pain or edema, hypercholesterolemia, and difficulties in ambulation) between January to December 2019. Patients who had already received a diagnosis of PAD or had undergone any prior lower limb revascularization procedures were excluded from the study. Data regarding patients’ demographics, comorbidities, previous surgical procedures, and medical history were collected from the hospital dataset and stored in an institutional database.

### 2.2. Ultrasound Scan of the Lower Limb and Patient Categorization

**Figure 1:**
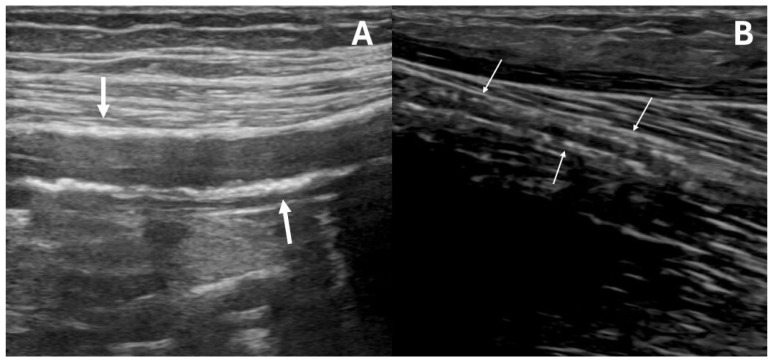
Ultrasound appearance of below-the-knee medial arterial calcification (MAC). MAC can appear as multiple hyperechogenic spots within the arterial wall (early stage, (**A**)) or as a continuous, hyperechogenic “railway” (late stage, (**B**)), as shown by the white arrows. The ultrasound image was magnified threefold (×3) and focused on its upper portion.

DUS scans were performed by the same experienced operator; the clinician was not involved in any eventual subsequent decision-making process, in the case of PAD or MACE development. All examinations were executed following a standardized protocol, according to the guidelines of the Society for Vascular Ultrasound for lower extremity arterial duplex examination [15] and employing the same US machine (LOGIQ S8 with XDclear, GE HealthCare Technologies Inc., Chicago, IL, USA) with a linear probe (frequency range 7–12 MHz). Both lower extremities were scanned: femoro–popliteal and BTK vessels were analyzed in transverse and longitudinal views. As a standard of care in our center, both B-Mode and color duplex evaluations were applied to acquire morphological and hemodynamic waveform characteristics, respectively [16,17]. MAC was diagnosed in the longitudinal view when multiple hyperechogenic spots (Figure 1A) or a calcified “railway” (Figure 1B) pattern were observed within the medial arterial layer, extending for at least 2 cm in the middle or distal segment of one or more BTK vessels [18]. These localizations, particularly the distal tracts (as shown in Supplementary Figures S2 and S3), were selected to ensure a uniform MAC assessment across patients, as evaluating the proximal segment of these vessels can be challenging, for example in subjects with a high Body Mass Index. According to the presence or absence of BTK MAC, patients were assigned to the MAC+ (MAC presence) or MAC− (MAC absence) cohort.

### 2.3. Outcomes

Long-term clinical data were obtained from the Regional Health Service Registry; all medical records updated over a 5-year period after the lower limb DUS scan were analyzed. Data collection for the outcomes stopped on 21 December 2024. The primary outcome was the rate of major adverse cardiovascular events during the follow-up period. MACE were considered nonfatal stroke, nonfatal myocardial infarction, and cardiovascular death, according to the American Heart Association [19,20]. The secondary outcomes included the incidence of new PAD diagnosis, defined according to the European Society for Vascular Surgery Guidelines [13], the rates of lower limb revascularization, including both endovascular and surgical procedures, major amputations, and death from any cause. Data were gathered from the Emilia–Romagna region dataset and were eventually censored at the date of death.

### 2.4. Statistical Analysis

Categorical variables were expressed as absolute values (*n*) and percentages (%). Variables with symmetric distribution were presented as mean and standard deviation (SD). The data distribution was verified using the Kolmogorov–Smirnov test. To assess differences in baseline characteristics between the two groups, the appropriate test (Chi-squared, Student’s *t* or Mann–Whitney) was employed according to the data distribution. With the chi-squared test, we verified the incidence of outcomes in the two cohorts. Kaplan–Meier estimates the time distribution from enrollment to the date of MACE, and a log-rank test for trend was used to compare the curves of the two groups. To avoid possible baseline imbalance between the two groups, a propensity score-matched analysis was employed. The selected covariates were the ones that were significantly different in the baseline comparison between the two groups. Using the Multivariate Cox proportional hazards regression analyses we analyzed the effects of several predictor variables on the primary outcome for each group. Owing to the limited number of events, multivariate hazard ratios (HR) were calculated using a forward approach, with an entry limit of *p* < 0.05. All the statistical analyses were performed employing Med-Calc Statistical Software version 23.0.2 (MedCalc Software Ltd., Ostend, Belgium). A *p* value < 0.05 was considered statistically significant.

## 3. Results

A total of 729 arterial lower limb ultrasound exams were executed during the study period. A cohort of 589 patients met the inclusion/exclusion criteria and was further analyzed; however, 5 patients had to be excluded due to the absence of any data on the medical records in the years of follow-up, so 584 patients were considered for the analysis. Here, 239 patients were assigned to the MAC+ group, while the remaining 345 subjects were included in the MAC− cohort. The demographic and baseline characteristics of all patients included in the study are reported in Table 1. According to the univariate analysis, the MAC+ group was younger (*p* < 0.001) and had a higher prevalence of diabetes mellitus (*p* < 0.001) and chronic kidney disease (*p* = 0.048). Instead, in the MAC− cohort, hypertension (*p* = 0.037), prior myocardial infarction (MI) (*p* < 0.001), stroke (*p* = 0.034), and smoking (*p* < 0.001) were more common.

Considering the differences in age and other risk factors, which could negatively affect the primary outcome of the study, a propensity score matching analysis for demographics and clinical characteristics was performed. After matching, a total of 324 patients, 162 for each cohort (MAC+ and MAC−), were included, as reported in Table 2.

### 3.1. Primary Outcome

**Figure 2:**
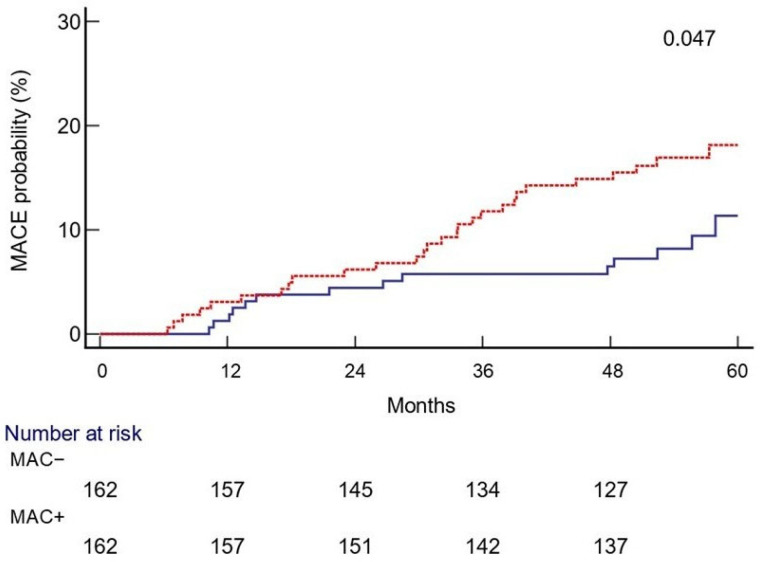
Probability of major adverse cardiovascular events (MACE) during the 5-year follow-up in the MAC + (red line) and MAC− (blue line) groups. Legend: Medial arterial calcification (MAC).

The median follow-up time was 57 months (interquartile range 53−60). During the follow-up, a total of 28 MACE occurred in the MAC+ group, whereas 14 events occurred in the MAC− cohort (*p* = 0.028). Moreover, MAC+ patients presented an almost two-fold (1.8) increased risk of MACE with respect to the other cohort [HR: 1.84; 95% CI: 1.01–3.38; *p* = 0.047] as shown in Figure 2. In the multivariate Cox regression analysis, age (HR 1.06; 1.01–1.12) and the presence of MAC (HR 1.22; 1.06–1.57) were identified as independent factors, associated with a greater risk of MACE.

### 3.2. Secondary Outcomes

**Figure 3:**
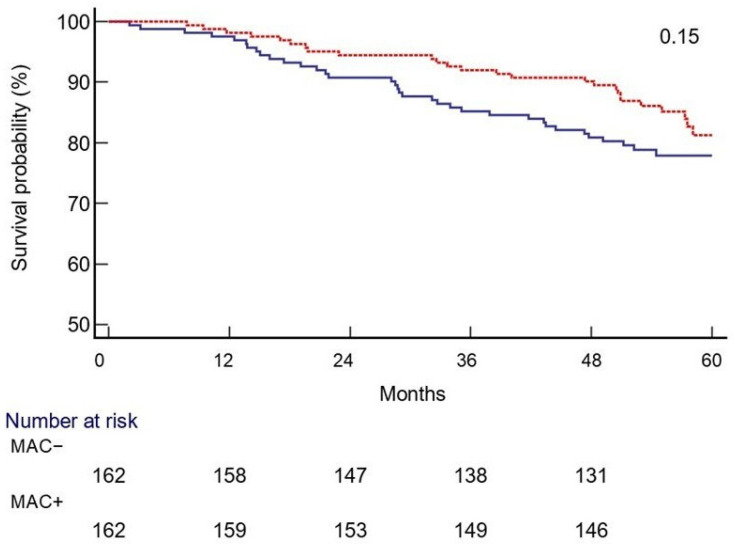
Survival probability during the 5-year follow-up in the MAC+ (red line) and MAC− (blue line) groups. Legend: Medial arterial calcification (MAC).

A total of 61 deaths occurred during the follow-up period, 26 and 35 in the MAC+ and MAC− groups, respectively (*p* = 0.24). A non-significantly increased all-cause mortality risk was observed in the MAC− cohort (HR: 1.43; 95% CI 0.86–2.37, *p* = 0.15), as shown in Figure 3. New PAD diagnoses occurred in 95 patients, 16 vs. 79 in the MAC+ and MAC− cohorts, respectively (*p* < 0.001); however, a limited and non-significant number of lower limb revascularization procedures occurred (*n* = 7), four in the MAC+ cohort and three in the MAC− group (*p* > 0.99), and no major amputations were registered in any group.

## 4. Discussion

A comparison of the two cohorts’ demographics at baseline revealed that the group with MAC (41% of the total) was significantly younger (*p* < 0.001), without differences between the two sexes. The true prevalence of MAC in the general population is not defined or the results are unknown in multiple districts (coronary arteries or thoracic aorta) because of the absence of a standardized methodology for detection and misdiagnosis with the atherosclerotic process. Some studies have reported a prevalence of MAC in the lower extremities of 2.5% (range 1.6–10%) in the general population, mainly in males, based on data collected from the ankle-brachial index (ABI) [21]. However, a few studies, with small sample sizes, have directly evaluated the correlation between ABI and MAC, supporting the conclusions of Hoek et al., that the presence of an elevated ABI is an insufficient proxy for MAC [22].

Radiological imaging, which is based on XRs, was the first systematic method applied to evaluate MAC [23]; for example, Ferraresi et al. developed the pedal MAC score (pMAC) to describe the severity of inframalleolar disease, using two-view XRs of the foot [8]. A high score obtained with this tool has been associated with a significant risk of major adverse limb events and major amputations [7,9,24] in CLTI patients. Nevertheless, these studies employed radiation and focused on high-risk symptomatic patients; the latter represents a substantial divergence in the population considered for the analysis and could explain why we did not highlight any differences in major adverse limb events (MALE) and amputations between our cohorts. In this context, US scanning of peripheral arterial vessels could represent a non-invasive, harmless technique for MAC evaluation [18].

In 2012, Liu et al. analyzed the presence of MAC in the femoral arteries of patients planned for amputation and established its relationship with diabetes [25], data that have been confirmed in our study (*p* < 0.001) and other studies [26,27,28]. Moreover, the higher prevalence of CKD (*p* = 0.048) in the MAC+ group was also highlighted by our analysis [4,6,29].

On the other hand, in the MAC− cohort, we found a significant prevalence of hypertension (*p* = 0.037) and smoking (*p* < 0.001), known risk factors associated with both carotid and coronary atherosclerotic plaque development [30,31]. In addition, analyzing the past medical history of this group, a significant number of patients with prior MI (*p* < 0.001) and stroke events (*p* = 0.034) were highlighted; this finding could be associated with the older age of this cohort and the aforementioned significant presence of risk factors, which are mainly linked to the intimal atherosclerotic process rather than to the development of medial arterial calcification. Moreover, age and MAC presence were independently associated with MACE events after propensity score matching analysis. Our findings comply with previous reports; at the end of the last century, several authors [5,10] associated radiological MAC detection with MACE in patients with type 2 DM, whereas Salle et al. [11] recently confirmed this link, basing on DUS scans of tibial vessels in an asymptomatic type 2 diabetic cohort. However, to our knowledge, no studies have focused on examining the prevalence of ultrasound MAC in tibial vessels in the general population, without focusing on a specific cluster of patients; the absence of similar studies, employing the same imaging and considering patients asymptomatic for PAD, hampers comparison with our findings.

An analysis of the secondary outcomes revealed non-significant differences in the number of total deaths and all-cause mortality risk between the two cohorts, but this data could be associated with the short follow-up period considered. During follow-up, instead, a significant number of PAD diagnosis afflicted MAC− cohort (*p* < 0.001); as highlighted by the ESVS guidelines [32], some risk factors, such as smoking, were more strongly correlated with atherosclerotic disease in the iliac–femoral axis rather than in the infrapopliteal district [33]. It is well known that the level where the lesion develops influences the emerging of PAD symptoms, as the progressive stenosis and final occlusion of the iliac axis or superficial femoral artery can be compensated for by the development of collateral vessels. These patients are usually asymptomatic or may present mild intermittent claudication, whose first management is the best medical therapy and supervised exercise program [13], with a risk of disease progression to CLTI between 5% and 10% during a 5-year period [32]. These data, along with the presence of a Vascular Rehabilitation Program to which patients were referred after diagnosis, can explain why, despite the number of new PAD diagnoses, the MAC− group did not undergo a significantly greater number of revascularization procedures or major amputations during follow-up [34].

### Study Strenghts and Limitations

One of the limitations of this study is the single-center collection of data and analysis. Information about the amount of packs/year in active or former smokers; the duration of DM, CKD, or hypertension; and the number of patients who received antivitamin K or cholecalciferol and bisphosphonate treatments, which are significant inducers of MAC [6], were not available in our study.

The operator dependence of DUS can represent another main issue; however, as highlighted in the methods section, all the examinations were performed by the same long time experienced vascular surgeon to reduce the impact of intervariability between operators. The use of US, nonetheless, can be noted as a key strength of this research: it represents a non-invasive, widely accessible and routinely employed imaging for tibial vessels evaluation, and allows the healthcare operator to evaluate MAC during the US exam session, avoiding further prescriptions of invasive diagnostic tests such as XRs in asymptomatic patients.

Several studies [7,8,9] have reported that infrapopliteal MAC, particularly in pedal arteries, is associated with major amputation in CLTI patients or major cardiovascular events in diabetic patients [11]. For this reason, we focused on analyzing below-the-knee and not above-the-knee vessels, in the middle and distal segments of the tibial vessel, which are widely accessible with the US evaluation.

Another limitation is that MAC evaluation was performed just once, and no further assessments, associated with possible clinical changes, were available. From a different point of view, the results obtained from this “one-time” DUS scan, executed on asymptomatic patients presented to our clinic for multiple and heterogeneous diagnostic indications (e.g., hypercholesterolemia or nonspecific lower-limb pain), could provide a broader overview of the potential impact of MAC on the general population.

In addition, the role of calcifications in pedal arteries and the relationship between the pMAC score and MALE have been clarified recently and were not available at the time of the study, so we did not perform any evaluation of MAC in the foot arteries. Finally, the 5-year follow-up period may not have been long enough to demonstrate a correlation between MAC presence and MALE, as highlighted by other studies [11], but could represent a warning for the healthcare operators in implementing medication management and strategies (e.g., prescribing further cardiac investigations) to prevent MACE.

## 5. Conclusions

In a cohort of asymptomatic patients, early detection of MAC in BTK vessels, through a non-invasive imaging modality such as DUS, was associated with a 1.8-fold increased risk for MACE through a 5-year period of follow-up but was not related to all-cause mortality risk, new PAD diagnosis, major amputations, or revascularization procedures. This aspect should be taken into account by all health care operators when performing DUS examinations on asymptomatic patients to optimize prevention strategies and medical therapy before the onset of MACE.

## Figures and Tables

**Table 1:** Baseline characteristics of the two study cohorts, MAC+ and MAC−, defined as the presence or absence of medial arterial calcification (MAC) in the tibial arteries, respectively, before the propensity-score matching analysis.

	All Patients (*N* = 584)		
Variables	MAC+ *N*, (%)	MAC− *N*, (%)	*p* Value
N. of patients	239 (41)	345 (59)	
Demographics			
Age (years ± SD)	73 ± 10	77 ± 11	<0.001 *
Male sex	134 (56)	208 (60)	0.31
Medical History			
Hypertension	200 (84)	309 (90)	0.037 *
Diabetes mellitus	183 (77)	162 (47)	<0.001 *
Hyperlipidemia	137 (57)	211 (62)	0.35
Smoking (ever)	78 (33)	166 (48)	<0.001 *
Chronic kidney disease	145 (61)	182 (53)	0.048 *
Prior MI	24 (10)	81 (24)	<0.001 *
Prior stroke	9 (4)	28 (8)	0.034 *
Rheumatological disorders	51 (21)	63 (18)	0.36
Former cancer diagnosis	41 (17)	66 (19)	0.78

Legend: Medial arterial calcification (MAC); standard deviation (SD); myocardial infarction (MI). The * indicates *p* values < 0.05

**Table 2:** Propensity score matching analysis for demographics and comorbidities between the groups: MAC presence (MAC+) and MAC absence (MAC−) in the tibial arteries.

Variables	MAC+ (*N* = 162) *n*, (%)	MAC− (*N* = 162) *n*, (%)	*p* Value
Demographics			
Age (years ± SD)	76 ± 8	76 ± 9	0.90
Male sex	97 (60)	104 (64)	0.42
Medical History			
Hypertension	151 (93)	151 (93)	>0.99
Diabetes mellitus	109 (67)	111 (69)	0.81
Hyperlipidemia	92 (57)	107 (66)	0.090
Smoking (ever)	92 (57)	85 (53)	0.21
Chronic kidney disease	95 (59)	95 (59)	>0.99
Prior MI	20 (12)	20 (12)	>0.99
Prior stroke	5 (3)	5 (3)	>0.99
Rheumatological disorders	39 (24)	28 (17)	0.13
Former cancer diagnosis	24 (15)	29 (18)	0.44

Legend: Medial arterial calcification (MAC); standard deviation (SD); myocardial infarction (MI).

## Data Availability

The dataset of the study is available upon request at bldgli@unife.it or nicola.lamberti@unife.it.

## Supplementary Materials

The following supporting information can be downloaded at https://www.mdpi.com/article/10.3390/diagnostics15172273/s1. Figure S1: STROBE flowchart. Figure S2: Image that illustrated the way to position the linear probe to allow the MAC visualization in longitudinal view at the level of the distal segment of the anterior tibial artery. Legend: Medial malleolus (M). Figure S3: Image that illustrated the way to position the linear probe to allow the MAC visualization in longitudinal view (behind the medial malleolus) at the level of the distal segment of the posterior tibial artery. Legend: Medial malleolus (M).

## Abbreviations

The following abbreviations are used in this manuscript:

ABI	Ankle-brachial index
BTK	Below-the-knee
CAD	Coronary artery disease
CI	Confidence interval
CKD	Chronic kidney disease
CLTI	Chronic limb-threatening ischemia
CT	Computed tomography
DUS	Duplex ultrasound
ECM	Extracellular matrix
DM	Diabetes mellitus
HR	Hazard ratio
MAC	Medial arterial calcification
MAC+	Medial Arterial Calcification presence
MAC−	Medial Arterial Calcification absence
MACE	Major adverse cardiovascular events
MALE	Major adverse limb events
MI	Myocardial infarction
LE	Lower extremity
PAD	Peripheral arterial disease
pMAC	Pedal medial arterial calcification
SD	Standard deviation
US	Ultrasound
XR	X-Rays
