# Supplementary figures and images for: Ultrasound Detection of Below-the-Knee Medial Arterial Calcifications in Asymptomatic Patients Is an Early Negative Predictor of Major Adverse Cardiovascular Events
Source: Diagnostics (Basel). 2025 Sep 8;15(17):2273. doi: 10.3390/diagnostics15172273 (PMC12427726; doi:10.3390/diagnostics15172273)

# Figure S1 - STROBE FLOWCHART

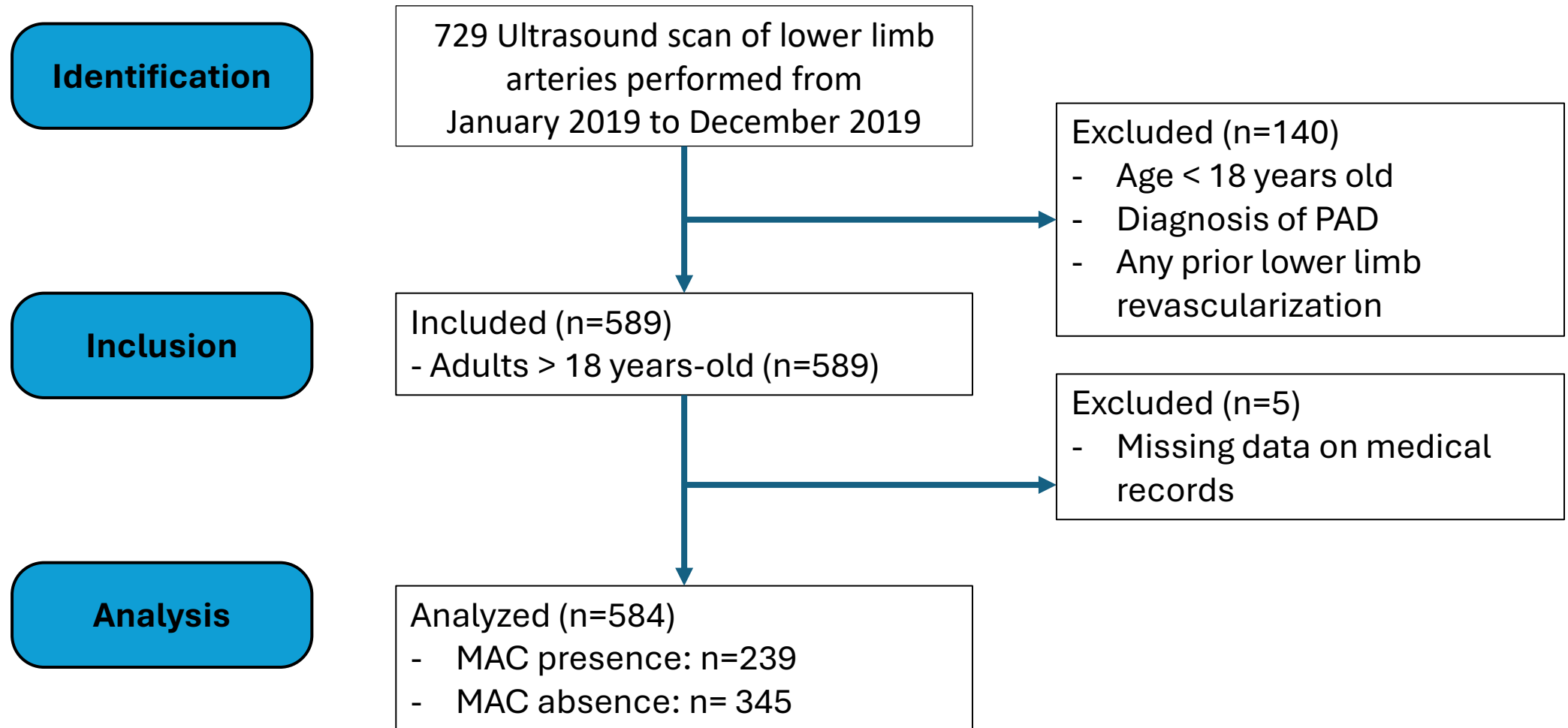

**Figure S2**

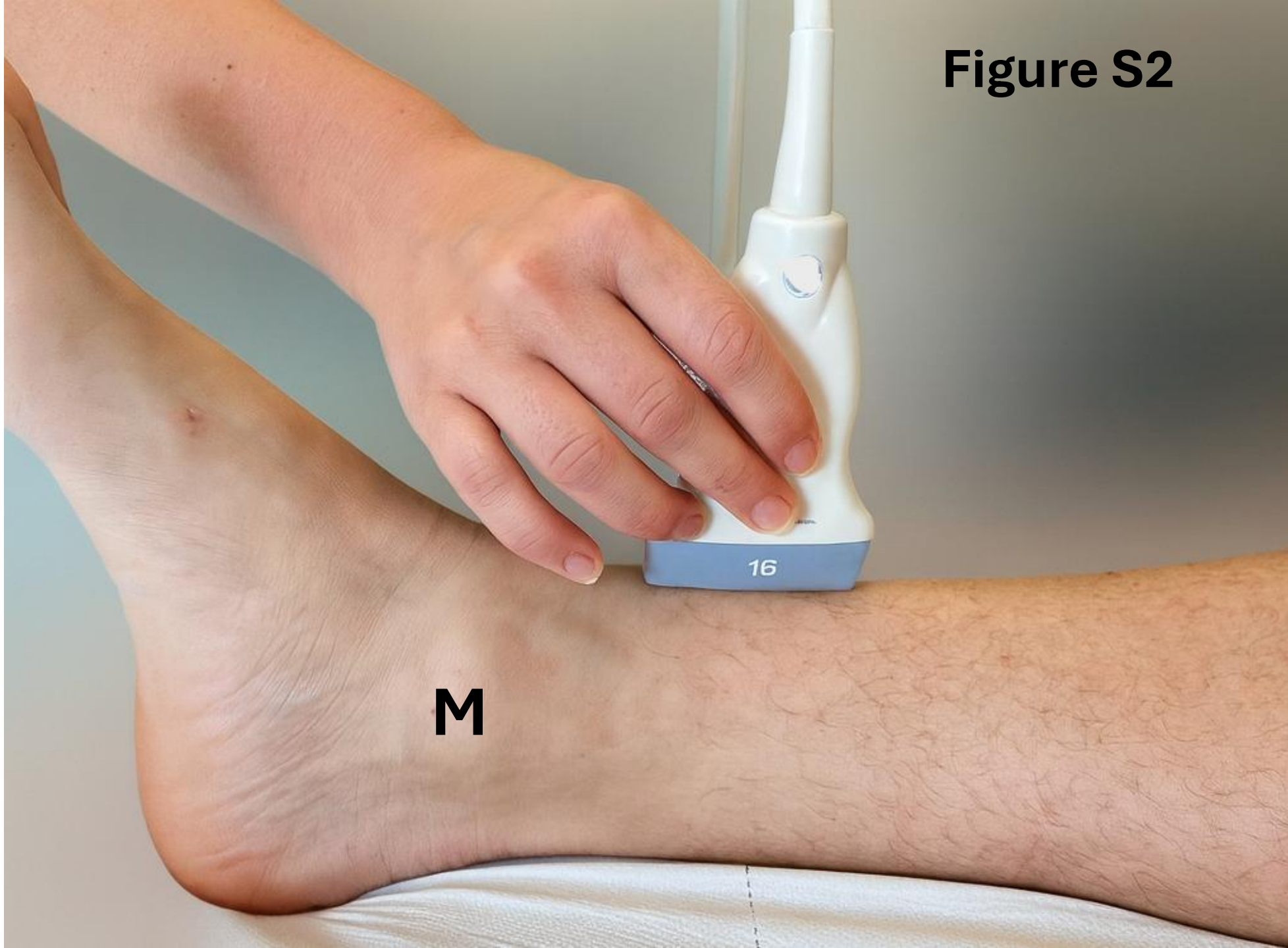

**Figure S3**

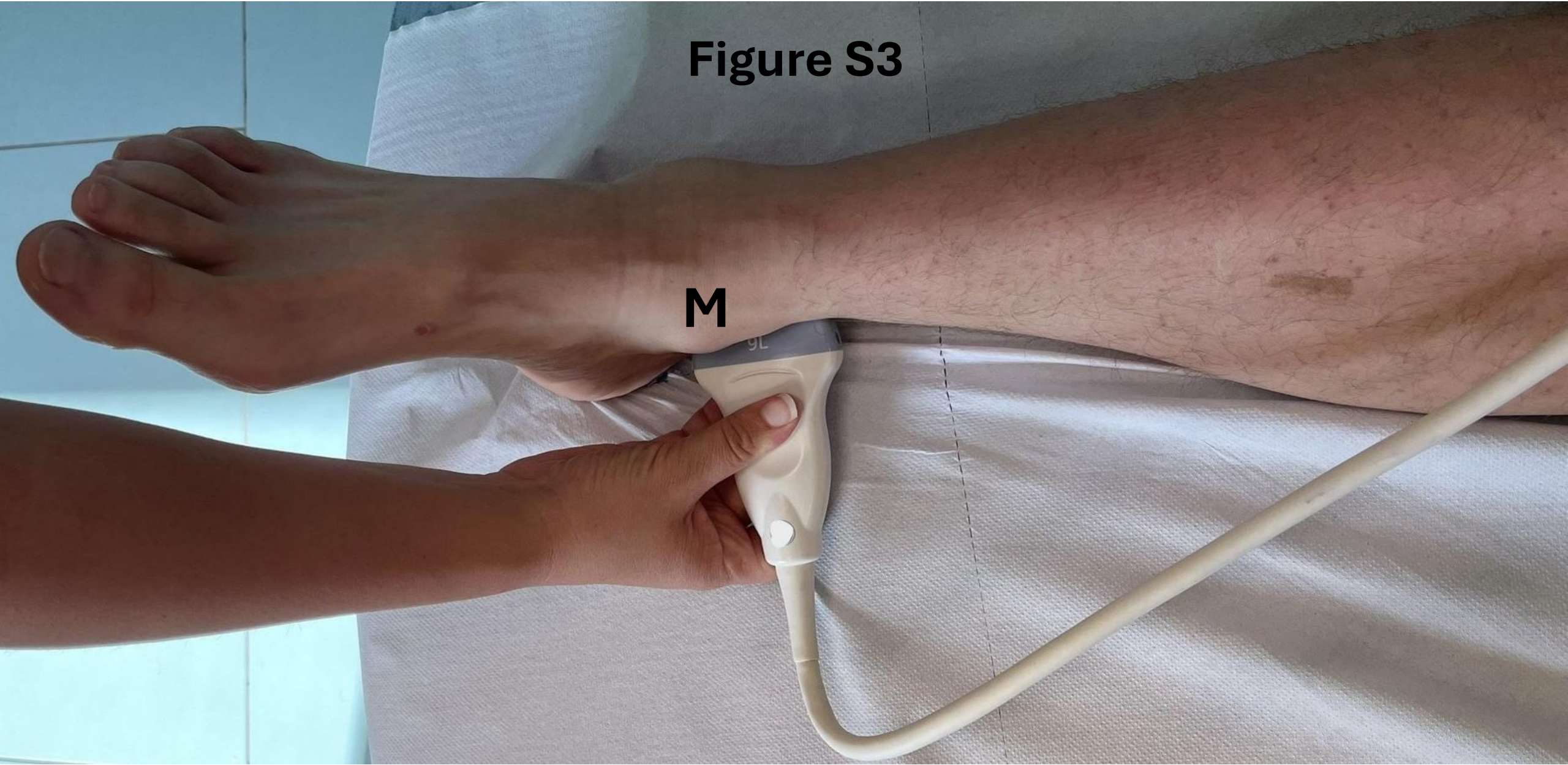

Supplement: Supplementary file 1 [file diagnostics-15-02273-s001.zip › diagnostics-3815527-supplementary.pdf]
